# Brain fingerprinting: let’s focus on the science—a reply to Meijer, Ben-Shakhar, Verschuere, and Donchin

**DOI:** 10.1007/s11571-012-9238-5

**Published:** 2013-01-09

**Authors:** Lawrence A. Farwell, Drew C. Richardson

**Affiliations:** 1 Brainwave Science/Brain Fingerprinting Laboratories, Government Works, Inc., 257 Turnpike Road, Suite 220, Southborough, MA 01772 USA; 2 FBI Laboratory (at the time of the research), 314 High Meadow Lane, Greenville, VA 24440 USA

**Keywords:** Brain fingerprinting, P300-MERMER, P300, Event-related potential, Concealed information test, MERMER

## Abstract

Farwell in Cogn Neurodyn 6:115–154, (2012) reviewed all research on brainwave-based detection of concealed information published in English, including the author’s laboratory and field research. He hypothesized that specific methods are sufficient to obtain less than 1 % error rate and high statistical confidence, and some of them are necessary. Farwell proposed 20 brain fingerprinting scientific standards embodying these methods. He documented the fact that all previous research and data are compatible with these hypotheses and standards. Farwell explained why failure to meet these standards resulted in decrements in performance of other, alternative methods. Meijer et al. criticized Farwell in Cogn Neurodyn 6:115–154, (2012) and Farwell personally. The authors stated their disagreement with Farwell’s hypotheses, but did not cite any data that contradict the three hypotheses, nor did they propose alternative hypotheses or standards. Meijer et al. made demonstrable misstatements of fact, including false *ad hominem* statements about Farwell, and impugned Farwell’s motives and character. We provide supporting evidence for Farwell’s three hypotheses, clarify several issues, correct Meijer et al.’s misstatements of fact, and propose that the progress of science is best served by practicing science: designing and conducting research to test and as necessary modify the proposed hypotheses and standards that explain the existing data.

## Summary of the science in Farwell (2012): three hypotheses and 20 scientific standards

Farwell (2012) reviewed all of the available literature published in English on detection of concealed information with event-related brain potentials. The most striking feature of the data is that there is a sharp bimodal distribution of error rates and statistical confidences. One set of methods, exemplified by Farwell and Donchin (1991); Farwell and Smith (2001); Farwell et al. (2012), related publications, and independent replications, always has produced less than 1 % error rate and high statistical confidences. Alternative methods, exemplified by Rosenfeld et al. (2004, 2008) and Miyake et al. (1993) have produced over 10 times higher error rates, and statistical confidences averaging 50 % (chance) for information-absent (“innocent”) determinations (Rosenfeld et al.). Farwell specified the fundamental differences in methods that have produced these large differences in results as 20 scientific standards, and defined brain fingerprinting as the technique embodying these specific standards.

Farwell advanced three hypotheses to account for all data existing to date:

### Hypothesis 1

Applying methods that substantially meet the 20 brain fingerprinting scientific standards provides sufficient conditions to produce less than 1 % error rate^1^ overall and less than 5 % error rate in every individual study. This holds true (1a) without countermeasures, (1b) with countermeasures, and (1c) in field cases where it is unknown whether countermeasures are being practiced or not.

### Hypothesis 2

Applying scientific methods that substantially meet the 20 scientific standards provides sufficient conditions to consistently produce statistical confidences for individual determinations, both information-present and information-absent, of at least 90 % for information-present determinations and 70 % in the opposite direction for information-absent determinations. (Farwell et al. (2012) increased this to 95 % for both.)

### Hypothesis 3

Some but not all of the 20 scientific standards are also necessary conditions to simultaneously obtain the above described levels of (3a) error rate and (3b) statistical confidence. The standards that are not necessary are nevertheless useful in that they improve accuracy and/or statistical confidence.

## Meijer et al.’s (2012) discussion of science in Farwell (2012): Farwell’s three hypotheses and 20 standards

Meijer et al. (2012) stated that Farwell’s hypotheses are not supported by the literature, but did not cite any relevant data in support of this position, nor did they propose alternative hypotheses to explain the existing data.

### Hypotheses 1 and 2

Meijer et al. (2012) stated that Hypothesis 1 (which they phrased in terms of “accuracy”)^2^ “stands in sharp contrast with the available literature.” They did not cite a single study, published or not, or even a single anecdotal case, in support of this statement. Farwell (2012) reviewed all of the relevant literature, and documented the fact that every case in every study supports Hypothesis 1: all studies that substantially met the defining 20 brain fingerprinting scientific standards achieved less than 1 % error rates, along with high statistical confidences for individual determinations.

How could Meijer et al.’s (2012) demonstrable and unequivocal misstatement of fact have arisen? Fundamentally different, alternative methods that may at first glance appear similar to brain fingerprinting have produced very different results. Rosenfeld et al. (2004), a purported replication of Farwell and Donchin (1991), produced more than 10 times higher error rates than those specified in Hypothesis 1 and achieved by Farwell and Donchin—in some conditions no better than chance accuracy—along with statistical confidences averaging 50 % (chance) for information-absent (“innocent”) subjects. So did Rosenfeld et al. (2008) and other subsequent “complex trial protocol” studies.

Why, then, do Miyake et al. (1993); Rosenfeld et al. (2004, 2008), and the other similar studies reviewed in Farwell (2012) not provide evidence against Hypotheses 1 and 2? Because Hypotheses 1 and 2 specify methods that meet the 20 brain fingerprinting scientific standards. Farwell explained in detail that Rosenfeld et al. and similar studies failed to meet even half of the 20 standards. Rosenfeld et al. (2004) is not anything close to a replication of Farwell and Donchin (1991). All studies that substantially met the 20 standards achieved the corresponding low (or in fact 0 %) error rates and high statistical confidences, as per Hypotheses 1 and 2.

Rosenfeld et al. (2004, 2008) and Miyake et al. (1993) do, however, provide support for Hypothesis 3: by failing to meet even half of the 20 standards and producing high error rates and low statistical confidences, they provide evidence that at least some of the 20 standards are necessary conditions for less than 1 % error rates and high statistical confidences.

Meijer et al. (2012) accuse Farwell (2012) of selection bias and “selectively dismissing relevant data” regarding Hypothesis 1. In fact, Farwell reviewed every relevant publication in English to date, and specified in detail the specific methodological differences that resulted in higher error rates and lower statistical confidences in the studies that failed to meet the 20 standards. He did not dismiss or ignore any relevant data.

Meijer et al. (2012), by contrast, dismissed all of the relevant data—which as Farwell comprehensively showed are all compatible with the three hypotheses—and did not cite a single study or case that was incompatible with Farwell’s hypotheses. Meijer et al. also engaged in selection bias. For example, they cited Rosenfeld (2005), an article critical of brain fingerprinting and of Farwell personally that contained several misstatements of fact. Several of these misstatements had been previously published and, when the publishers later checked the facts and realized the statements were false, they published corrections. Farwell (2011a) published a reply in the same journal that corrected Rosenfeld’s misstatements of fact and presented Farwell’s opposing views along with supporting data, documentation, and references. Meijer et al. cited only Rosenfeld’s paper, and not Farwell’s.

Meijer et al. (2012) postulate the existence of “studies demonstrating that brain fingerprinting is sensitive to countermeasures,” but do not cite any such studies. They cannot cite any such studies, because no such studies exist. Nor do they cite any anecdotal evidence, or any supporting data at all. Farwell discussed every published paper on countermeasures, and described in detail the methodological shortcomings in the techniques that were shown to susceptible to countermeasures and how these techniques were fundamentally different from brain fingerprinting.

No one has ever beaten a brain fingerprinting test with countermeasures (or without countermeasures), despite life-or-death motivation in field cases and a $100,000 reward for doing so (Farwell 2012; Farwell et al. 2012). The countermeasures that proved effective against Rosenfeld et al.’s (2004, 2008) methods and other methods had no effect on brain fingerprinting (Farwell et al. 2012).

As Farwell (2012, p. 150) stated, “To be meaningful and practically useful, generalizations about brainwave-based concealed information tests must distinguish between the studies that meet the brain fingerprinting standards and those that fail to meet the standards. Generalizations that fail to recognize this distinction are inadequate to present a meaningful interpretation of the available data, and can result in drawing erroneous conclusions about brain fingerprinting that in fact apply only to non-brain fingerprinting tests that fail to meet the standards. For example, the low accuracy and susceptibility to countermeasures characteristic of several non-brain fingerprinting techniques has sometimes been erroneously generalized to apply to brain fingerprinting, whereas in fact the actual data directly contradict this generalization.”

### Hypothesis 3

Meijer et al. (2012) question Hypothesis 3 based on Farwell and Donchin (1991). They point out that Farwell and Donchin did not meet standards 4, 8, and 10, and nevertheless achieved the same 0 % error rate as the other brain fingerprinting studies. This does not contradict Hypothesis 3, which states that some, and not all, of the 20 standards are necessary. Standards 4, 8, and 10 are not necessary. They are refinements we developed in response to the challenges of field applications. Our current working hypothesis is that standards 4, 8, and 10, although not necessary, do nevertheless improve error rate and/or statistical confidence. These three standards may explain why the methods of Farwell and Donchin produced 12.5 % indeterminates, whereas studies that met all 20 standards have produced 0 % indeterminates as well as 0 % error rate in all research to date (e.g., Farwell and Smith 2001; Farwell et al. 2012).

### P300 and P300-MERMER

Meijer et al. (2012) question whether the P300-MERMER has any incremental value beyond the P300 alone. This is a valid and important scientific question. Seven studies address this question (see Farwell 2012 for a review). Due to overriding security concerns, however, publication of full details of several of our studies at the FBI, the CIA, and the US Navy has previously not been possible. At the time of Farwell (2012), only one relevant study, Farwell and Smith (2001), had been published in full form in a peer-reviewed journal. The security concerns are now resolved, we have recently published three peer-reviewed papers, and several more are under review or in preparation.

Four studies that directly address this question have now been fully peer-reviewed and published (Farwell et al. 2012). (At the time of Farwell 2012, these studies had been published only as abstracts.) These four field/real life studies compared P300 and P300-MERMER in the detection of concealed information regarding (1) real-life events including felony crimes; (2) real crimes with substantial consequences (either a judicial outcome, i.e., evidence admitted in court, or a $100,000 reward for beating the test); (3) knowledge unique to FBI agents; and (4) knowledge unique to explosives (EOD/IED) experts.

All 76 determinations (74 individuals) were correct with both the P300-MERMER-based analysis and the P300-based analysis. P300-MERMER provided higher statistical confidences than P300 in a majority of subjects. This difference between P300 and P300-MERMER was highly statistically significant in each of the four studies. The data to date support the hypothesis that the P300-MERMER has incremental value beyond the P300 alone. Moreover, Farwell (2012) reviewed extensive evidence from intra-cranial recordings demonstrating that the voltage pattern of the P300-MERMER occurs not only at the scalp but also in relevant brain structures, which supports the validity of the P300-MERMER as a neurophysiological phenomenon. In our view, further research comparing the P300 and P300-MERMER will be valuable.

### The 20 brain fingerprinting scientific standards

Meijer et al. (2012) do not provide any evidence or data contrary to Farwell’s three hypotheses regarding the 20 standards, nor do they suggest any modifications to the standards or any alternative standards. Meijer et al. state “These twenty standards, however, represents (sic) merely Farwell’s subjective views…” Recall what the standards actually entail, e.g., “Instruct the subjects to press one button in response to targets…”; “Use a mathematical classification algorithm…” The standards are simply a set of methods. They are purely objective.

Farwell does express a subjective view, specifically that to be viable for field use a set of methods must produce error rates of less than 1 % along with high statistical confidences. The 20 standards constitute an objective statement of the methods that, so far, have produced such results. Meijer et al. (2012) present no evidence contrary to this fact. Perhaps the word “methods” would have been more appropriate than “standards.” In any case, Farwell’s (or anyone else’s) subjective feelings and opinions about these methods are beside the point. Their value rests solely in the results they have consistently produced. Future research may, of course, demonstrate that refinement or modification of these methods will minimize error rate and/or maximize statistical confidence.

## Other, non-scientific issues raised by Meijer et al. (2012)

Meijer et al. (2012) state, “Interestingly, the University of Illinois patented the original P300 based CIT as published in Farwell and Donchin (1991). And conveniently, the ‘discovery’ and patenting of the MERMER liberates him (Farwell) from the constraints of this earlier patent.” This statement, besides being irrelevant to the scientific issues at hand, is unequivocally and demonstrably false. According to patent law, a prior patent takes precedence over any future patent. Everything in the prior patent remained unaffected by Farwell’s four subsequent US patents. To obtain additional patents, Farwell had to prove to the satisfaction of the United States Patent and Trademark Office (USPTO) that his new discovery of the P300-MERMER was “novel, useful, and non-obvious” over the state of the prior art, including the University of Illinois patent and the P300. What “liberates” Farwell from the previous patent was not his new patents, but rather the fact that the University of Illinois failed to pay the maintenance fee, so the USPTO ruled the patent abandoned.

Meijer et al. (2012) appear to advocate an absolute taboo against mentioning in a scholarly work anything that has not been previously published in a peer-reviewed journal. We disagree. Other types of publications are published because they are perceived to have merit and can provide relevant and useful data and insights. Patents require proof that the patented invention is “novel, useful, and non-obvious.” Book chapters and encyclopedia entries are scrutinized by knowledgeable editors. Doctoral dissertations pass muster with committees of experts. Conference abstracts have some measure of scrutiny by editors. In the context of Farwell (2012), Farwell’s relevant previous publications include not only six previous peer-reviewed scientific papers (Farwell 2011a; Farwell and Donchin 1988, 1991; Farwell et al. 1993; Farwell and Smith 2001; Rapp et al. 1993),^3^ but also a dissertation (Farwell 1992), two book chapters (Donchin et al. 1986; Miller et al. 1987); an encyclopedia entry (Farwell 2013), five patents (Farwell 1994, 1995a, b, 2007, 2010), a legal publication (Farwell and Makeig 2005), a monograph (Farwell 2011b), and several conference abstracts (e.g., Farwell, Richardson, and Richardson 2011). Moreover, since Farwell (2012), Farwell and colleagues have published four additional relevant studies (see Farwell, Richardson, and Richardson 2012).

Among the authors of the two papers under discussion here (Farwell 2012; Meijer et al. 2012), Farwell is not the only one to include discussion of such sources in his scholarly writings. Meijer et al. (2012) cite and meaningfully discuss Farwell and Donchin (1986), a conference abstract. In book chapters authored by both Farwell and Donchin, along with others (Donchin et al. 1986; Miller et al. 1987) the authors discuss in considerable detail the methods, results, and relevant data on P300, memory, and aging published previously in Farwell et al. (1985), a conference abstract—and not published in full form in a peer-reviewed journal. Applying an absolute taboo against discussing such data only to Farwell (2012) and not to the writings of others—such as the authors of Meijer et al.—would be discriminatory and inconsistent, and would not serve the best interests of readers who would like to know the full story.

Moreover, fortunately, the discussion of the distinction between conference abstracts and peer-reviewed publications has become largely moot with the recent peer-reviewed publication (Farwell et al. 2012) of four studies cited in Farwell (2012) as conference abstracts, and will become entirely moot with additional upcoming publications.

Meijer et al.’s (2012) table 1 represents the peer-reviewed publications and “verdicts”^4^ therein at the time of Farwell (2012). It does not, however, represent a complete picture of all of the relevant evidence on brain fingerprinting at that time, as discussed above. Moreover, since that time an additional four studies including 76 subject tests on 74 individuals have been published in a peer-reviewed journal (Farwell et al. 2012). (These were previously published as conference abstracts.)

Meijer et al. (2012) accuse Farwell of misrepresenting conference abstracts as full-fledged peer-reviewed publications. Consider the following. Farwell (2012) cited Farwell and Donchin (1986) as follows: “In the initial brain fingerprinting research, Farwell and Donchin used the P300 event-related brain potential (Farwell and Donchin 1986….” “Three types of stimuli are presented: probes, targets, and irrelevants. (Farwell & Donchin 1986…” “Farwell and Donchin (1986, 1991) made it clear that brain fingerprinting detects information, not lies, guilt, or actions.”

Meijer et al. cited the same publication as follows: “The variant of the CIT with ERPs was first investigated in the late 80ties [sic] (Farwell and Donchin 1986…” In the reference sections of the respective papers, the citations are identical word for word. Other citations in both papers are similar.

Meijer et al. (2012) provide no criterion by which they judge Farwell’s citations to be “misrepresenting” and their own to be clearly delineating the difference between conference abstracts and peer-reviewed papers. Our perspective is that the readers of *Cognitive Neurodynamics* are highly intelligent and knowledgeable. We presume them to be intelligent enough to follow Farwell’s discussion of the significance of intracranial recordings in the inferior parietal lobe/supramarginal gyrus, superior temporal sulcus, the amygdala and hippocampus, dorsolateral and orbital frontal cortices, and the anterior cingulate, and his discussion of the mathematical distinctions between bootstrapping classification and comparison algorithms and the resultant differences in statistical confidences. Such individuals, in our view, can find their way to the reference section and readily distinguish between different types of publications.

One clarification in terminology is in order. Meijer et al. (2012) point out a linguistic anomaly that might cause some confusion, and we would like to take this opportunity to clarify the situation. Consider the situation wherein John Smith is tested as a subject on a set of stimuli for which he is “information present,” and John Smith is also tested on a different set of stimuli for which he is “information absent.” How many information-present subjects are tested? One. How many information-absent subjects are tested? One. How many total subjects are tested? The answer “one” leads to the anomaly 1 + 1 = 1. The answer “two” is correct in terms of the number of tests run (and the statistical power of the design), but it is not quite correct in that two of the “subjects” were actually the same person. “Subject tests” may be a better term for avoiding ambiguity. The statement “There were two subject tests,” along with a disclosure of the experimental design in which the same participant was run as a subject in two different tests, provides a more complete and unambiguous account. The summary charts in Farwell (2012) (tables 2 and 3) used the word “subjects” to refer to the number of “subject tests” undertaken, even when one individual participated in more than one test. Substituting the column heading “subject tests” would be a useful change that would clear up any possible ambiguity.

Meijer et al. (2012) use the term “participants” to refer to individual human beings and “verdicts” to refer to subject tests. In our view “verdicts” is inappropriate, because brain fingerprinting does not deliver a legal verdict, but only detects information.^5^ We prefer the term “subject tests” for reasons described above.^6^ The term “participants” may also be ambiguous, as it may be construed to refer only to people who participated in a crime or mock crime, or to all participants in the research.

None of this is an issue for anyone familiar with the relevant literature, however, because in all of Farwell’s publications (e.g., Farwell and Donchin 1991), the number of tests and the number of people who participated have been clearly delineated, and the authors have clearly disclosed when one person is a subject in more than one test. Moreover, this makes no difference in the statistics computed or the scientific conclusions drawn from the data. Nevertheless, we are happy to provide a clarification as above.

Meijer et al. (2012) impugned Farwell’s motives and character, as follows. As is common in the field, Farwell and Donchin published their research first as a conference abstract (Farwell and Donchin 1986) and later as a full peer-reviewed paper (Farwell and Donchin 1991). Both Farwell (2012) and Meijer et al. cite and meaningfully discuss both of the Farwell and Donchin papers. Obviously, Farwell’s comprehensive tutorial review includes more detail than Meijer et al.’s brief communication in the discussion of these and other papers. Farwell includes tables 2 and 3, which present the number of subject tests in the various studies discussed (see above discussion on terminology). Meijer et al.—and not Farwell—added together the numbers of subjects in the various studies such that the numbers were duplicated. That is, when two publications [an abstract and a subsequent full paper such as Farwell and Donchin (1986, 1991)] reported on the same research, Meijer et al. counted the tests twice, thus inflating the totals for field and laboratory tests. Thus the totals Meijer et al. computed for laboratory and field studies do not reflect the actual total numbers of individuals or subject tests in the studies. Then, on the basis of their own misrepresentative addition—which does not appear in Farwell (2012)—and/or on the basis of some difference they postulate but do not describe in the manner of citing the respective papers (see above discussion), Meijer et al. accuse Farwell of “deliberately duplicating participants and studies.”

We will leave it to the readers of *Cognitive Neurodynamics* to form their own judgments as to whether either, both, or neither of Farwell’s (2012) and Meijer et al.’s (2012) writings constitute “duplicating participants and studies.” We choose not to speculate on the motives of our fellow scientists, so we will not address the question of whether Meijer et al.’s actions in this regard were “deliberate” or not. In any case, in our view, Meijer et al.’s impugning of Farwell’s motives and character does not advance the progress of science.

In any case, none of this changes the fundamental scientific issues at hand or the scientific conclusions warranted by the data. The progress of science is driven by research and data, not by words. Any way you name, rename, misname, parse, count, recount, miscount, discount, or don’t count the publications, people, and tests, the fact remains that all available data (including Meijer et al.’s table 1) are compatible with Hypotheses 1–3 and the 20 scientific standards proposed in Farwell (2012).

Meijer et al. (2012) made a number of other *ad hominem* comments about Farwell, his motives, character, subjective state, intentions, behavior, writing style, etc. In our view, further discussion of such matters will not serve the progress of science or the interests of our readers.

Meijer et al. (2012) state that Farwell and colleagues implemented standard 4 for “some unexplained reason.” Standard 4 specifies using situation-relevant (or crime-relevant) targets, rather than inherently irrelevant targets made relevant only by instructions. In fact, Farwell (2012) explained in detail their reasoning and the considerable value of situation-relevant targets in reference to the FBI agent study, devoting 536 words and one figure to this. Farwell et al. (2012) explained this in even more detail.

Farwell (2012, pp. 118–122) devoted 4,005 words to a comprehensive discussion of the functional significance, antecedent conditions, history, neurodynamics, physiological mechanism, and signal characteristics P300 and P300-MERMER. Meijer et al. (2012) quoted one sentence of this out of context, represented it as Farwell’s view of the P300, and criticized it as being inadequate. We agree that this sentence, or virtually any other single sentence from Farwell or any other publication, is an inadequate description of the P300.^7^ We encourage readers to read Farwell’s full article.

Meijer et al. (2012) state that the “P300-MERMER… is unlikely to solve the problem caused by the lack of a one-to-one relationship between P300 and memory.” Neither Farwell nor, to our knowledge, anyone other than Meijer et al. has suggested that there is (or should be) a one-to-one relationship between P300 and memory, or considered the lack of such a relationship to constitute a “problem” to “solve.” Again, we encourage readers to read Farwell’s (2012) entire article for a comprehensive discussion of the P300 and P300-MERMER and their role in the detection of concealed information.

## Correction

We have documented misstatements of fact in Meijer et al. (2012). To be fair, we must acknowledge that there was also one error in Farwell (2012). Although this made no difference in the statistics computed or the scientific conclusions, we take this opportunity to correct it.

Farwell (2012) documented the fact that the methods of Rosenfeld et al. (2004, 2008) and subsequent studies resulted in average statistical confidences no better than chance (50 %) for information-absent subjects, and that half of the statistical confidences reported for information-absent subjects were actually less than chance. That is, some subjects were (correctly) classified as information absent (“innocent”) when according to the statistics computed there was less than 50 % probability that this determination was correct, and greater than 50 % probability that the opposite (information present/“guilty”) determination was correct. This is in accord with the predictions of the statistical model applied, and also with the actual data when reported. This is factually correct information provided by Farwell and supported by the relevant publications cited. A footnote (Farwell p. 147, footnote 4), however, gave an incorrect example of this from a prior publication. The corrected footnote, providing a correct example of this phenomenon, reads as follows:

For example, in Meixner et al. (2009, p. 215), Table 2, “innocent” subject 11, the subject was determined to be ‘‘innocent’’ when the computed probability was 85 % that ‘‘guilty’’ was the correct determination (i.e., that the probe P300 was larger than the irrelevant P300, which is the definition of “guilty” in the “Iall” condition). Statistical confidence for this (correct) determination was 15 %, far less than chance. Six of 10 subjects correctly determined to be “innocent” in this condition had statistical confidences of less than 50 % (chance) that this determination was correct.

## Let’s focus on the science

In our view, the progress of science is best served by actually practicing science. With respect to the subject matter at hand, this means designing and conducting scientific studies to test the three hypotheses and 20 scientific standards that have up until now proven to be compatible with all known research results and data.

Meijer et al. (2012) stated their disagreement with Farwell’s hypotheses, but provided no data that contradicted the three hypotheses or the 20 standards. Nor did they propose alternative hypotheses or standards to explain the existing data.

These three hypotheses and 20 standards are not, in our view, the final answer. They are simply the only proposed explanation that currently fits all the data and accounts for the existing bimodal distribution in error rates and statistical confidences. Future research and data may of course require modifications, additions, subtractions, or substitutions in the three hypotheses and 20 standards, or for that matter their complete replacement with a better explanation of the new data discovered in the future. Our job as scientists is to practice the relevant science and to conduct the relevant research.

In our view, we as scientists have a responsibility not just to satisfy the curiosity of other scientists, but more importantly to serve the public. For those of us who practice brain fingerprinting in the field, peoples’ lives and freedom depend on this science, and on its being practiced with the most effective methods available.

This is not merely an academic consideration for the victims of serial killer JB Grinder, or for whoever would have been his next victim had he not been put in prison with the help of brain fingerprinting, or for Terry Harrington, who was in prison for 23 years before brain fingerprinting was ruled admissible in his case. It is not merely an academic consideration for the victims of terrorists and serial killers who are still out there getting away with murder, or for other innocents like Harrington who are still falsely imprisoned. Human lives and well-being depend on the progress of this science and its effective application in the real world.

In our view, scientific progress is best served not by a war of words (and certainly not by impugning the motives and character of other scientists) but rather by conducting new scientific research in the laboratory and the field; reporting the results thereof; and revising our hypotheses, theoretical understanding, and methods as necessary in the light of these new findings. Scientific progress and human life are also served by applying the best available scientific methods to address the needs of people whose lives can benefit from this science. In our view, focusing on the actual practice of science is the best and only viable path forward, not only for the sake of scientific progress, but more importantly for the sake of all of those whose lives and well-being now and in the future depend on this science being practiced as effectively as possible.

## Footnotes

1 To date, studies that meet the brain fingerprinting standards have produced 0 % error rate. We characterize this as “<1 %” to provide a conservative estimate and to avoid the mathematical anomalies of 0 %.

2 We use “error rate” rather than “accuracy,” because error rate is the prevailing legal standard, and error rate has a single unambiguous definition, while accuracy is defined differently by different authors.

3 All six of these, incidentally, meet the recommendations of Simmons et al. (2011).

4 What they call a “verdict” Farwell has called a “subject,” and would probably better be called a “subject test” as described below. “Verdict” is a legal term that is inappropriate here, because brain fingerprinting detects information, not legal culpability.

5 Farwell and Donchin’s (1991) experiment 1 subjects were “innocent” or “guilty” of a mock crime, but were actually tested on information, not guilt. We now use the more accurate terms “information present” and “information absent.”

6 The term “cases” also refers to tests, and includes field tests that were not undertaken in the course of a scientific study, and consequently wherein individuals may not be considered experimental subjects. Many cases are reported in reports on criminal investigations, court proceedings, legal publications, etc., rather than in scientific journals.

7 For example, Meijer et al.’s (2012) one-sentence summary (“…the P300 is elicited by any event that violates the subject’s expectancies”) is equally inadequate: it addresses subjective probability, but fails to address task relevance, an equally important factor.
